# Body weight and high‐fat diet are associated with epigenetic aging in female members of the BXD murine family

**DOI:** 10.1111/acel.13207

**Published:** 2020-08-12

**Authors:** Jose Vladimir Sandoval‐Sierra, Alexandra H. B. Helbing, Evan G. Williams, David G. Ashbrook, Suheeta Roy, Robert W. Williams, Khyobeni Mozhui

**Affiliations:** ^1^ Department of Preventive Medicine University of Tennessee Health Science Center College of Medicine Memphis TN USA; ^2^ Department of Genetics, Genomics and Informatics University of Tennessee Health Science Center College of Medicine Memphis TN USA; ^3^ Luxembourg Centre for Systems Biomedicine University of Luxembourg Esch‐sur‐Alzette Luxembourg

**Keywords:** age acceleration, DNA methylation clock, life span, longevity

## Abstract

DNA methylation (DNAm) is shaped by genetic and environmental factors and modulated by aging. Here, we examine interrelations between epigenetic aging, body weight (BW), and life span in 12 isogenic strains from the BXD family of mice that exhibit over twofold variation in longevity. Genome‐wide DNAm was assayed in 70 liver specimens from predominantly female cases, 6–25 months old, that were maintained on normal chow or high‐fat diet (HFD). We defined subsets of CpG regions associated with age, BW at young adulthood, and strain‐by‐diet‐dependent life span. These age‐associated differentially methylated CpG regions (age‐DMRs) featured distinct genomic characteristics, with DNAm gains over time occurring in sites such as promoters and exons that have high CpG density and low average methylation. CpG regions associated with BW were enriched in introns, tended to have lower methylation in mice with higher BW, and were inversely correlated with gene expression (i.e., higher mRNA levels in mice with higher BW). CpG regions associated with life span were linked to genes involved in life span modulation, including the telomerase reverse transcriptase gene, *Tert*, which had both lower methylation and higher expression in long‐lived strains. An epigenetic clock defined from age‐DMRs revealed accelerated aging in mice belonging to strains with shorter life spans. Both higher BW and the HFD were associated with accelerated epigenetic aging. Our results highlight the age‐accelerating effect of heavier BW. Furthermore, we demonstrate that the measure of epigenetic aging derived from age‐DMRs can predict genotype and diet‐induced differences in life span among female BXD members.

## Graphical abstract

The epigenetic clock, also referred to as DNA methylation age, provides a measure of biological aging. This clock can predict life expectancy in genetically distinct mouse stains, and is accelerated by higher body weight and high‐fat diet.

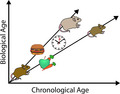

## 1 INTRODUCTION

The “epigenetic clock” based on DNA methylation (DNAm) has emerged as a widely used biomarker of aging that surpasses telomere length assays in its accuracy and utility (Breitling et al., 2016; Marioni et al., 2018). Often referred to as DNAm age (DNAmAge), the CpG‐based estimator of biological age comes in a few different versions for both humans and mice (Hannum et al., 2013; Horvath, 2013; Levine et al., 2018; Petkovich et al., 2017; Stubbs et al., 2017; Thompson et al., 2018; Wang et al., 2017). All these clocks share a common feature—they rely on the methylation status of preselected subsets of CpGs that are each assigned weights and are used collectively to estimate age. A critical question has been: are these DNAmAge clocks detecting changes that are purely a function of time, and therefore, correlates of chronological age? Or are they providing a measure of the intrinsic pace of biological aging that can be related to health, fitness, and life expectancy? Evidence from human epidemiological studies indicates that some versions of the clock perform well at predicting life expectancy, and a younger DNAmAge relative to chronological age implies decelerated biological aging, and is associated with a lower risk of disease and increased longevity (Chen et al., 2016; Levine et al., 2018; Lu et al., 2019; Marioni, Shah, McRae, Chen, et al., 2015; Marioni, Shah, McRae, Ritchie, et al., 2015).

In mice, life span extending interventions such as calorie restriction (CR) and treatment with rapamycin, or strong genetic mutations that drastically reduce body weight (e.g., *Ames* and *Snell* dwarf mice), have been shown to significantly decelerate the epigenetic clock (Cole et al., 2017; Petkovich et al., 2017; Sziraki, Tyshkovskiy, & Gladyshev, 2018; Thompson et al., 2018; Wang et al., 2017). However, to date, most DNAmAge estimations in mice have been modeled on the canonical C57BL/6J (B6) background strain (Cole et al., 2017; Petkovich et al., 2017; Stubbs et al., 2017; Thompson et al., 2018; Wang et al., 2017). Similar to interindividual variation in humans, aging trajectories vary considerably among different mouse strains, and common DNA variants contribute to the pace of normal aging and longevity (Yuan et al., 2009). Whether the differential rates of epigenetic aging can discern normative life span differences between mouse strains remains an open question. Furthermore, if body weight reduction due to CR or dwarfing mutations can slow down the clock, then another question is: can subtle differences in normal body weight also have an impact on DNAmAge?

The BXD recombinant inbred strains are the most deeply phenotyped mouse genetic reference panel, and have a long history in aging and longevity research (De Haan & Van Zant, 1999; Gelman, Watson, Bronson, & Yunis, 1988; Haan, Gelman, Watson, Yunis, & Van Zant, 1998). Notably, the different BXD sibling strains exhibit wide variation in life expectancy. Some strains have a mean life expectancy of less than 15 months (e.g., BXD13, BXD5), while others typically live well over two or even 3 years (e.g., BXD19, BXD65) (Gelman et al., 1988; Haan et al., 1998; Lang et al., 2010; Roy et al., 2019). Longevity data in the BXDs have been collected since the 1980s. Life span data continue to be collected from an enlarged family that now consists of 150 sets of isogenic siblings (Ashbrook et al., 2019; Gelman et al., 1988; Roy et al., 2019). The BXDs were derived by crossing two parental strains, B6 and DBA/2J (D2), and then inbreeding the progeny (Ashbrook et al., 2019). Genomes of the BXDs therefore represent random recombinations of the B6 and D2 genomes, and each strain is a unique mosaic of homozygous B6 or D2 genotypes. The D2 strain is considered to have a more accelerated aging profile, and has consistently shorter life span than B6 (Yuan, Peters, & Paigen, 2011; Yuan et al., 2009). Other age‐associated parameters include rapid thymic involution (Hsu, Li, Zhang, & Mountz, 2005), quicker replicative senescence of hematopoietic stem cells (De Haan & Van Zant, 1999), and increased tail tendon breakage in D2 compared to B6 (Sloane et al., 2011). Due to random assortment of gene variants, the progeny BXDs have a greater range of variation in life span and aging traits (De Haan & Van Zant, 1999; Roy et al., 2019), and provide a unique resource with which to dissect the interrelations between epigenetic aging and longevity.

Here, we leveraged the extensive longevity data generated for the BXDs (Roy et al., 2019) to evaluate associations between body weight, epigenetic aging, and life span. We used affinity‐capture enrichment with the methyl‐CpG‐binding domain protein (MBD), followed by deep sequencing (MBD‐seq) to profile the aging liver methylome of 12 BXD strains (Aberg, Chan, Xie, Shabalin, & van den Oord, 2018). To examine the impact of a common metabolic stressor, we also quantified the methylome on a subset of BXD cases maintained on a high‐fat diet (HFD), a diet that decreased life span by as much as 12% (Roy et al., 2019). Our goal was to chart differentially methylated regions related to aging, and to strain differences in body weight and life span, and to examine correlation with gene expression. We computed a simple DNAm clock using the age‐dependent CpG regions, and this revealed strain differences in rates of epigenetic aging. Overall, our results show that both higher body weight and HFD can have an accelerating effect on the epigenetic age, and this can then be related to life span.

## 2 RESULTS

### 2.1 BXD strain selection and life span and body weight characteristics

**FIGURE 1:**
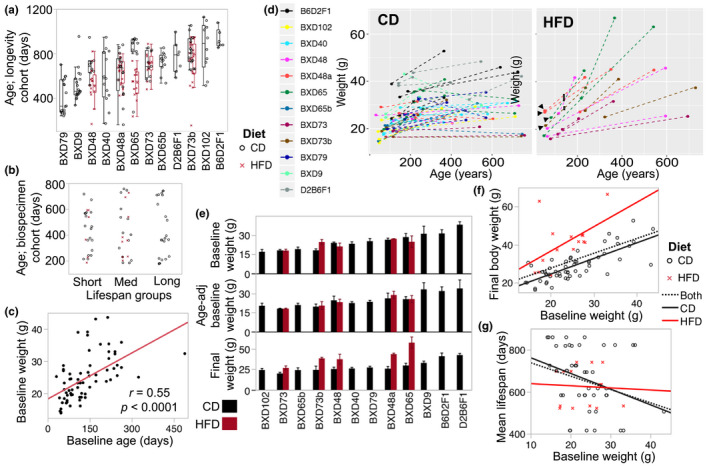
Age distribution, and life span and body weight characteristics. (a) Each point depicts a *longevity cohort* mouse that was allowed to age till mortality either on control diet (CD; black circles) or high‐fat diet (HFD; red crosses). There was a total of 225 mice, and mean sample size was 13 (ranging from 6 to 22) per strain‐by‐diet. (b) Each point depicts a *biospecimen cohort* mouse used for tissue harvest and methylome assay. Age distribution (*y*‐axis) is uniform across the three life span groups. (c) At baseline, before introduction to HFD, age was significantly correlated with body weight. (d) Individual trajectory of change in body weight from baseline to final age is plotted with each mouse colored by strain. Mice in the HFD group were introduced to the diet at the time of initial weight measurement, with the exception of 4 mice belonging to BXD48a, BXD65, and BXD73b (black arrows) that were placed on HFD at age ~145 (entry to HFD marked by black rectangles for the 4 mice). (e) The bar plots show average body weight at young adulthood (original baseline weight, and residual values after adjustment for age), and final body weight. Error bars are standard error. (f) The baseline body weight (*x*‐axis) was a significant predictor of individual body weight at older age (*y*‐axis); *r* = 0.53 (*p* < 0.0001) for all mice; *r* = 0.77 (*p* < 0.0001) CD group; *r* = 0.53 (*p* = 0.04) for 15 HFD group. (g) Baseline weight was negatively correlated with mean life span (*y*‐axis) for the strain‐by‐diet groups. However, when separated by diet group, this inverse correlation was significant only for the CD group; *r *= –0.27 (*p* = 0.04) for all mice; *r *= –0.31 (*p* = 0.04) for the CD group; ns for HFD groups

The present work is based on data collected from two separate cohorts of BXD mice. Life span data were collected from a group of female BXDs, referred to as the “*longevity cohort*,” that were kept either on *ad libitum* standard chow (control diet or CD) or on HFD, and allowed to age until mortality. The BXD strains exhibited a wide range in natural life span, and HFD reduced overall longevity. Details on this *longevity cohort* are reported in Roy et al. (2019). Based on the life span data, 12 members of the BXD panel (including F1 hybrids) were selected for DNAm assays as they were representative of the wide variation in life span. This included related sub‐strains with differences in life span (BXD65/BXD65b, and BXD73/BXD73b; Table 1 and Figure 1a). Figure 1a plots the ages at natural death for mice in the *longevity cohort*, and sample sizes for life span determination for these selected strains range from 6 to 22. For five of these, we included mice maintained on HFD as these strains showed significant reduction in life span (specifically, BXD65 on HFD; Figure 1a) or were related sub‐strains with variable response to HFD (e.g., BXD48 had slight life span reduction, but BXD48a appeared unaffected by HFD; Table 1). Based on life span averages, each strain‐by‐diet group was classified as short‐lived (mean life span <600 days), medium‐lived (600–750 days), and long‐lived (>800 days; no strain on HFD were in this group). Note that a strain classified as long‐lived on CD may be classified as short‐lived on HFD (Table 1).

**TABLE 1:** Characteristics of selected strains from the BXD family.

Strain/line	Mean life span (days)^a^	Longevity trait			Diet^c^	Biospecimen	
		Median life span (days)^a^	Range (days)^a^	Life span Group^b^		*N* ^c^	Age range (days)^c^
B6D2F1	933 ± 86	896	856–1080	Long	CD	5^d^	216–726
BXD102	861 ± 222	891	514–1131	Long	CD	5^d^	183–714
BXD40	585 ± 239	577	172–949	Short	CD	8	284–719
BXD48	695 ± 124	684	516–948	Med	CD	3	188–731
BXD48	523 ± 152	517	167–821	Short	HFD	3	189–595
BXD48a	617 ± 196	670	161–881	Med	CD	3	233–604
BXD48a	635 ± 113	650	424–800	Med	HFD	3	233–543
BXD65	824 ± 199	896	275–938	Long	CD	6	181–711
BXD65	534 ± 128	551	236–736	Short	HFD	3^e^	230–541
BXD65b	726 ± 91	751	536–854	Med	CD	4	187–748
BXD73	702 ± 116	687	553–854	Med	CD	4	206–759
BXD73	699 ± 112	715	480–889	Med	HFD	3	206–694
BXD73b	820 ± 129	807	656–1055	Long	CD	3	237–743
BXD73b	742 ± 193	790	154–951	Med	HFD	3	237–729
BXD79	417 ± 155	330	241–703	Short	CD	7	217–570
BXD9	507 ± 135	462	337–956	Short	CD	3	245–548
D2B6F1	771 ± 143	791	585–996	Long	CD	4^d^	210–744

^a^ Life span for strains under standard or high‐fat diet estimated from an aging cohort of mice co‐housed with mice used for biospecimen collection and methylome assays.

^b^ Groups based on phenotypic life span: short = average life span <600 days; med = average 640–750 days, and long = average life span >800 days.

^c^ Diet, sample size, and age range of mice used to generate methylome data. CD = control diet; HFD = high‐fat diet.

^d^ One male case; see Table S1 for individual‐level data.

^e^ One case excluded due to uncertain identity.

A parallel, identically treated and strain‐matched group, the “*biospecimen cohort*,” was maintained for tissue collection at different ages, and liver samples were obtained from this. We selected 70 liver specimens for the corresponding 17 strain‐by‐diet groups. These were chosen so that distribution of age at time of tissue collection was closely matched across the life span groups (Figure 1b; individual‐level sample information in Table S1). We note that aside from three male cases for BXD102, B6D2F1, and D2B6F1 (Table S1), all liver specimens were from females. Mice were initially weighed at young adulthood (mean age of 134 ± 81 days), and at this stage, age was a significant correlate of weight (Figure 1c). We refer to this as baseline body weight or BW0. The mice assigned to HFD were introduced to the diet at the time of initial weighing, with the exception of four that were introduced 90 days later (see Figure 1d). Mice spent 73–614 days on HFD (Table S1) before final weighing on the day of sample collection, by which time, the group on HFD had become significantly heavier to the matched strains on CD (HFD = 41 ± 12 g vs. CD = 26 ± 6 g, *p* < 0.0001; *n* = 34). For the HFD group, the final weight was uncorrelated with the number of days on HFD. The weight of the liver on HFD was slightly heavier but the effect was not statistically significant, likely due to the modest sample size for HFD (HFD = 1.29 ± 0.23 g vs. CD = 1.22 ± 0.23 g; *p* = 0.37, *n* = 34). There was significant strain variation in BW0 even after adjusting for age, and the long‐lived F1s in particular, had higher body weights, and this hybrid vigor was apparent with or without the male cases (Figure 1e). The final weight of mice also showed significant strain variation (Figure 1e). Liver weight appeared fairly consistent across strains. By final weighing, age was no longer a significant correlate of body weight (*r* = 0.01) or liver weight (*r* = 0.12). Instead, BW0 remained a significant predictor of final weight in both the CD and HFD mice (Figure 1f). We performed a multivariable regression to evaluate the degree to which the final body weight was predicted by BW0, diet, strain, and chronological age. This showed that the strongest predictors of final weight were diet (*F*_(1,55)_ = 61, *p *= <0.0001), followed by baseline weight (*F*_(1,55)_ = 7, *p* = 0.009), and strain (*F*_(11,55)_ = 3, *p* = 0.0095), but not the final age of mice.

We next examined correlations between the individual weight measures from the *biospecimen cohort* and strain‐level life span from the *longevity cohort*. The F1s exhibited vigor in both longevity and body weight and including the F1s resulted in no significant correlation between weight and life span. After excluding the F1s, both BW0 and final weight showed inverse correlations with all the strain‐level indicators of life span (mean, median, maximum, and minimum life span) (Table S2). When separated by diet group, the correlation between mean life span and BW0 was significant only for the CD group (Figure 1g). A multivariable regression with strain, BW0, and diet showed that only strain (*F*_(9,48)_ = 42, *p *= <0.0001) and diet (*F*_(1,48)_ = 45, *p *= <.0001), but not BW0 (*F*_(1,48)_ = 1.5, *p* = 0.2), were significant predictors of strain mean life span. Since BW0 is highly dependent on strain, the variance attributed to BW0 may have lessened after accounting for the effect of strain. However, a similar multivariable regression with strain, final weight, and diet showed that along with strain (*F*_(9,48)_ = 49, *p* = <0.0001) and diet (*F*_(1,48)_ = 8, *p* = 0.007), the final weight (*F*_(1,48)_ = 7, *β* = −2.9, *p* = 0.009) was also a significant predictor of mean life span. Overall, the results indicate that mice with higher body weight are more likely to belong to strains with shorter life span, and these observations are consistent with the strong inverse correlation between body weight and longevity that is seen in the larger BXD longevity panel (Roy et al., 2019).

### 2.2 Strain‐dependent patterns in global features of the methylome

Following genome‐wide MBD‐sequencing, we retained a set of 368,300 regions, each 150 bp in length, with sufficient coverage in the 70 samples. The majority of the CpG regions (83%) contained no sequence variants (SNPs or small insertions/deletions) segregating in the BXDs. For the 17% (62,422) with sequence variants, there was an average of 2 ± 1.6 variants within the 150 bp bin. Consistent with the DNA enrichment and filtering protocols, the 368,300 CpG regions were enriched in annotated gene features such as UTR, introns, exons and CpG islands, and also rRNA and LTRs compared to the background genome (Table S3).

**FIGURE 2:**
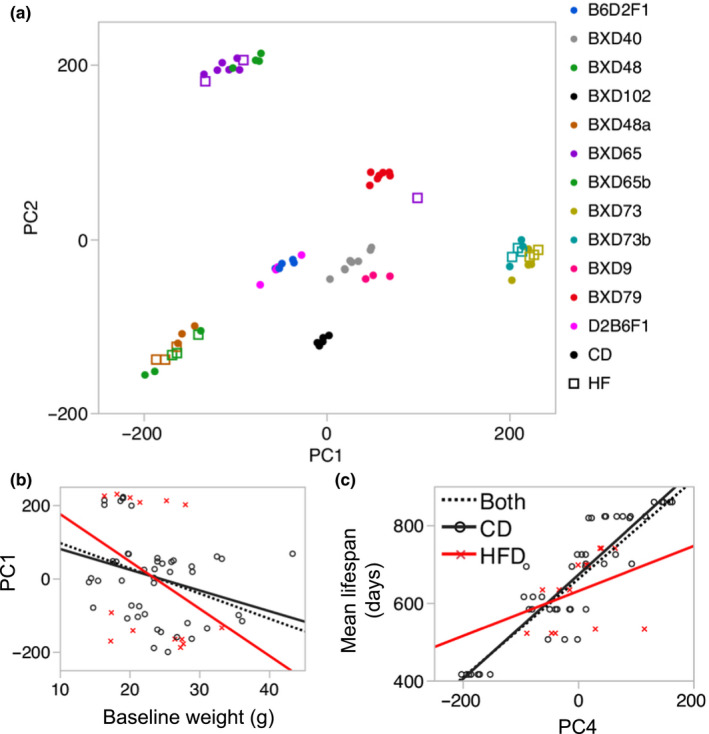
Global features of the methylome. (a) Scatter plot between the top 2 principal components—PC1 (19% of variance) and PC2 (13% of variance)—shows a strong population structure with mice clustering by strain identity (color coded). For strains with cases on both standard chow (CD; solid circles) and high‐fat diet (HFD; squares), the HFD and CD samples co‐cluster. (b) Body weight at young adulthood has a significant negative correlation with PC1 (*r *= –0.3, *p* = 0.02, *n* = 69). When partitioned by diet, the negative correlation is significant only for the CD (*r *= –0.30, *p* = 0.05). For HFD group, the negative correlation does not reach statistical significance (*r *= –0.35, *p* = 0.22). (c) The methylome in turn may be predictive of life span, and PC4 is strongly correlated with life span (*r* = 0.85, *p *= <0.0001, *n* = 69). When partitioned by diet group, the correlation is significant for the CD (*r* = 0.90; *p* < 0.0001), but not for the HFD group (*r* = 0.35; *p* = 0.22)

We started with a principal component analysis (PCA). A plot of the top principal components, PC1 and PC2 (captured 19% and 13% of the variance, respectively), showed clustering of samples by strain identity, irrespective of diet (Figure 2a). The one exception was a BXD65 on HFD that plotted away from the BXD65 cluster, and due to its questionable identity, it was excluded from downstream analyses, and remaining statistical tests were done in 69 samples. Sub‐strains (e.g., BXD73/BXD73b; BXD65/BXD65b) also clustered in close proximity. Unsupervised hierarchical clustering confirmed the clustering of samples by strain identity rather than age or diet groups (QC plots in Figure S1).

The top 5 PCs collectively explained 58% of the variance (Table S1), and we examined whether these were associated with age, diet, body and liver weights, and strain life span. Age was not a significant correlate of any of the 5 PCs. For strains with matched CD and HFD samples, the PCs did not differentiate between the diets. For the weight measurements, PC1 was a significant negative correlate of BW0 (Figure 2b), and final body and liver weights (correlations with and without F1s in Table S4). When partitioned by diet, the negative correlations remained significant only for the CD group. None of the other PCs were associated with the weight measurements. For the strain longevity data, PC4 (8% of variance) was the strongest correlate of mean, median, minimum, and maximum life span (Figure 2c; Table S4). PC2 and PC3 (11% of variance) were also significantly correlated with maximum life span (Table S4). These correlations between PCs and life span remained significant when restricted to the CD group.

We computed the overall mean methylation for genic regions (i.e., CpG regions that overlap annotated gene features), and intergenic regions, to examine whether these global features would explain the variance captured by the top PCs. Global methylation was highly strain specific (Figures S2a and S2b; Table S1), and the mean methylation of intergenic CpGs was significantly correlated with PC1 and PC3 (Figures S2c and S2d). Mean methylation of genic CpGs was significantly correlated with PC4. However, the mean methylation values were not directly correlated with the longevity traits. For the weight measurements, only the mean methylation of intergenic sites showed a consistent and significant positive correlation with liver weight (Table S4).

Taken together, the methylome‐wide analysis showed that the PCs capture strain‐dependent differences in overall mean methylation. These PCs were also associated with body weight, and strain‐level life span, but not age and diet.

### 2.3 Characterizing differentially methylated CpG regions

**FIGURE 3:**
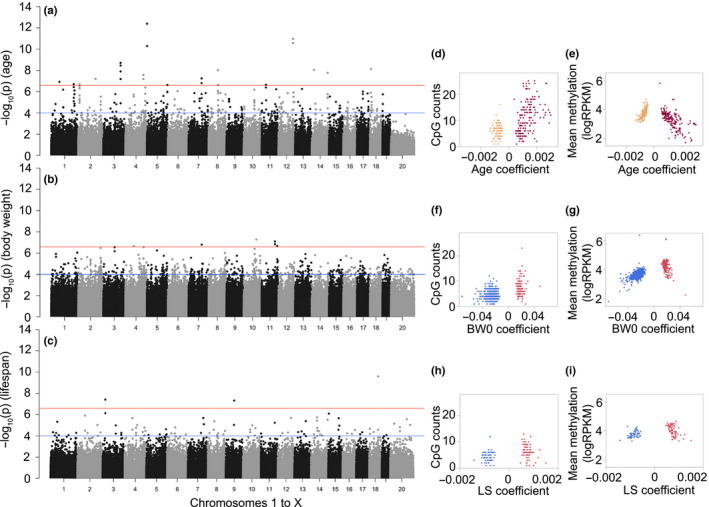
Features of differentially methylated CpGs regions (DMRs). Each point in the Manhattan plot represents the location of a CpG region (*x*‐axis: autosomal chromosomes 1–19, and chromosome X as 20), and the association –log_10_ *p* (*y*‐axis) for (a) age effect, (b) body weight at young adulthood (BW0), and (c) median life span (LS). The genome‐wide significant threshold is set at –log_10_(2.7e−7) (red line; 10% Bonferroni threshold for 368,300 tests) and the suggestive threshold at –log_10_(1.0e−4) (blue line). For the age‐DMRs, the regression coefficients for age (i.e., change in DNA methylation per unit change in age in days, log_10_ scale), and whether a site gained (positive coefficients, burgundy) or lost methylation (negative coefficients; sandy brown) was highly dependent on (d) the CpG density, and (e) mean methylation. For BW0‐DMRs, the body weight associated coefficients also showed correlations with the CpG counts within the bin (f), and sites that were negatively associated with BW0 (blue) had lower mean methylation than the sites that were positively associated with BW0 (red) (g). For the LS‐DMRs, whether a site was positively (red) or negatively (blue) associated with strain median life span was only modestly dependent on (h) the CpG counts, and not dependent on (i) the mean methylation levels

We next applied an epigenome‐wide association study (EWAS) approach to identify CpG regions that were associated with age, BW0, and strain median life span using a multiple linear mixed model. Age was a significant predictor of site‐specific DNAm (Figure 3a), and 26 regions, covering 319 CpGs, were above the 10% Bonferroni threshold (unadjusted *p* ≤ 2.7 × 10^−7^). These strong age‐dependent differentially methylation regions (age‐DMRs) were mostly located within genes, and 25 of the 26 bins were associated with increased methylation with age (age hypermethylation; Table S5). Although BW0 and the life span traits had strong associations with global DNAm patterns, after partly accounting for the strain‐dependent effects with the mixed model, only seven CpG regions were significantly associated with BW0 at the 10% Bonferroni threshold (Figure 3b). All these BW0 associated differentially methylation regions (BW0‐DMRs) had lower methylation among mice with higher BW0 (negative regression estimates; Table S6). At the same Bonferroni threshold, only three CpG regions were associated with strain‐level median life span and are referred to as LS‐DMRs (Figure 3c; Table S7).

Given the non‐independence of adjacent CpG regions, we then applied a lenient threshold of uncorrected *p* ≤ 1.0 × 10^−4^ to define the general characteristics of the sites associated with age, BW0, and life span. In total, 306 CpG regions were age‐DMRs at this suggestive threshold (Figure 3a; Table S5). Of these, 57% were age‐hypermethylated (Table 2). Compared to the background set of 368,300 CpG bins, the age‐DMRs were highly enriched in genic regions such as promoters and exons, and CpG island, and depleted in intergenic regions (enrichment and depletion *p*‐values in Table S3). For each age‐DMR, we computed the average methylation and CpG density, and compared these to the age regression coefficients, which convey the change as a function of age. The regression estimates appeared to be highly dependent on local genomic characteristics, and the most pronounced changes involved age hypermethylation (positive regression coefficients) in bins with high CpG density (Figure 3d) and lower average methylation (Figure 3e). In contrast, age‐DMRs that lost methylation with age (age‐hypomethylated; negative regression coefficients) featured lower CpG density, and higher average methylation (Figure 3d,e). Functional annotations of these regions identified significant enrichment in gene sets involved in establishment of cellular polarity (e.g., *Wnt5a*, *Lrrd1*, *Ptk7*) (Table S8). We consulted the human GWAS catalog to identify genes represented by the age‐DMRs that have been significantly associated with human aging and longevity (Table S5), and this identified only one gene, *Cux2* (Buniello et al., 2019).

**TABLE 2:** Tally of differentially methylated regions.

	Positive coefficients			Negative coefficients			Variants
	*N*	Genic	Intergenic	*N*	Genic	Intergenic	
Age‐DMR	175	146 (48%)	29 (9%)	131	94 (31%)	37 (12%)	51 of 306 (17%)
BW0‐DMR	172	93 (14%)	79 (11%)	517	368 (53%)	149 (22%)	137 of 689 (20%)
LS‐DMR	73	25 (20%)	48 (39%)	51	31 (25%)	20 (16%)	27 of 124 (21.77%)

At the suggestive threshold, 689 CpG regions were classified as BW0‐DMRs, and the majority of these (517 or 75%) were negatively correlated with BW0 (Table 2). Introns were the most enriched gene feature (Tables S3 and S6). Compared to the age‐DMRs, the BW0‐DMRs were in regions with relatively lower CpG density and had only 3 regions within CpG islands (Figure 3f). The regions that were negatively correlated with BW0 in particular featured lower CpG densities and also lower mean methylation levels (Figure 3g). Significantly enriched gene ontologies (GO) included regulation of protein homooligomerization, actin cytoskeleton reorganization, and GTPase‐mediated signal transduction (Table S8). We also note that several of these genes have been associated with weight in human GWAS, including *Hmga2*, *Fto*, and *Ntrk2* (Table S6) (Metrustry et al., 2014; Thorleifsson et al., 2009; Weedon et al., 2007).

For life span, there were 124 LS‐DMRs (Table S7), and a slight majority of these (59%, mostly intergenic sites) were positively correlated with higher median life span (Table 2). The LS‐DMRs did not show any enrichment or depletion in gene features compared to the background set, and the LS‐DMRs featured lower CpG density (Figure 3h). There was no difference in methylation levels between the DMRs that had positive or negative regression estimates (Figure 3i). The LS‐DMRs were not enriched in any particular biological GO category. However, there was a significant enrichment in genes that result in premature death in single knockout mice, and this included an intronic DMR in the telomerase reverse transcriptase gene (*Tert*) (Table S8). Also enriched were genes related to abnormal eating behavior and a decrease in body mass index (e.g., *Igfbp3*, *Mc4r*, *Lpar1*), and genes related to abnormal mineral levels (*Calcr*, *Sptb*, *Wwox*).

In terms of the potential effect of underlying sequence differences, we examined what fraction of the DMRs were sites that overlapped DNA variants segregating in the BXDs (Table 2). Since the background set has 62,422 bins with variants, the 51 variant containing age‐DMRs did not represent a biased enrichment of such regions (hypergeometric enrichment *p* = 0.58). The BW0‐DMRs and LS‐DMRs were only slightly enriched in sequence variants (enrichment *p* = 0.02 and *p* = 0.09, respectively) (Table 2).

### 2.4 Relating differentially methylated regions to gene expression

**FIGURE 4:**
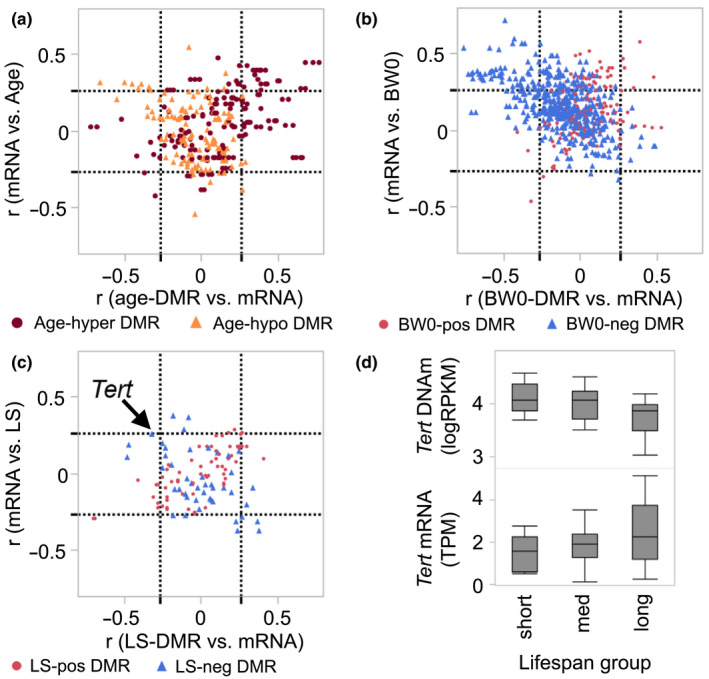
Comparison between DMRs and expression of cognate genes. (a) The plot shows correlations between the age‐DMRs and expression of corresponding transcripts (Pearson *r* on *x*‐axis), and the correlation between the transcript and age of mice (*y*‐axis). The dashed lines demarcate the nominally significant |*r*| = 0.27 threshold (*p* ≤ 0.05; *n* = 52 samples with matched MBD‐seq and RNA‐seq). Most of the age‐hypermethylated DMRs (burgundy circles) are positively correlated with mRNA, and these mRNAs also tend to be positively correlated with age (top right square of graph). A few of the age‐hypomethylated‐DMRs (sandy brown triangles) have negative correlation with gene expression, and the corresponding transcripts are positively correlated with age (top‐left square). (b) Majority of the BW0‐DMRs are negatively associated with BW0 (blue triangles), and most of the DMR‐mRNA pairs are located in the top‐left square of the plot, that is, gene expression is negatively correlated with DNA methylation and positively correlated with BW0. A few of the BW0‐DMRs that have positive associations with BW0 (red circles) also have positive correlations with gene expression (top right square). (c) The few LS‐DMRs have modest correlations with gene expression. The DMR for *Tert* is negatively correlated with transcript (arrow), and the transcript has modest correlation with median life span at *r* = 0.26 (*p* = 0.07). (d) Comparison of DNA methylation levels (top) in the three life span groups shows significantly lower methylation in the long‐lived group (*F*_2,66_ = 7.67, *p* = 0.001). A similar comparison for gene expression shows a significantly higher gene expression in the long‐lived group (*F*_2,49_ = 3,31, *p* = 0.04)

Fifty‐two of the samples with liver MBD‐seq also had matched liver RNA‐seq data, and we used this group to examine whether the DMRs were associated with gene expression. We linked the age‐, BW0‐, and LS‐DMRs to the corresponding transcript. Of the 306 age‐DMRs, 279 were paired with mRNA. For these pairs, we tested how many of the DMRs were *cis*‐correlated with gene expression, and how many of the transcripts were also correlated with age of mice (Table S9). At an uncorrected *p* ≤ 0.05 (|*r*| ≥ 0.27), 79 age‐DMRs were correlated with the expression of cognate genes (Figure 4a). The age‐hypermethylated DMRs mostly showed positive correlations with gene expression such that the transcripts also showed increased expression with age (e.g., *Jak3*, *Amn*, *Tradd*). The age‐hypomethylated DMRs were more likely to be negatively correlated with gene expression, and this set of transcripts also showed increased expression with age (*Nfkbia*, *Slit3*).

For the BW0‐DMRs, 614 of the CpG regions were paired to corresponding transcripts. At *p* ≤ 0.05 (|*r*| ≥ 0.27), 121 BW0‐DMRs were correlated with gene expression (Table S10). The overall pattern indicated that the DMRs that had lower methylation in mice with higher BW0 (i.e., negative regression estimates) tended to be negatively correlated with gene expression, and there was higher expression of these genes in mice with higher BW0 (Figure 4b). This included *Mc5r*, *Nfkb1*, and *Tcf4*. The few DMRs that had positive associations with BW0 were more likely to be positively correlated with gene expression (e.g., *Aldh18a1*, *Madd*, *Rap2a*).

For the LS‐DMR, 111 paired to corresponding transcripts, and only 19 CpG regions were *cis*‐correlated with gene expression (Figure 4c; Table S11). The strongest *cis*‐correlations were between the LS‐DMRs linked to *Hpse* and *Dapp1* (*r* = –0.70 for both). Both CpG regions had positive regression values for life span, and the corresponding transcripts had lower expression in mice belonging to strains with longer life span. The LS‐DMR in *Tert* was also negatively correlated with the expression of the *Tert* mRNA (Figure 4c, *r* = –0.32, *p* = 0.02), and the mRNA itself had a modest positive correlation with life span (*r* = 0.26, *p* = 0.07). Comparison of *Tert* methylation and gene expression among the three life span groups showed that the longer lived group had significantly lower methylation levels compared to both the medium‐ and short‐lived groups (*F*_2,66_ = 7.67, *p* = 0.001), and the long‐lived group had significantly higher gene expression compared to the short‐lived group (*F*_2,49_ = 3,31, *p* = 0.04; Figure 4d).

### 2.5 Measure of age acceleration from age‐associated CpG regions

**FIGURE 5:**
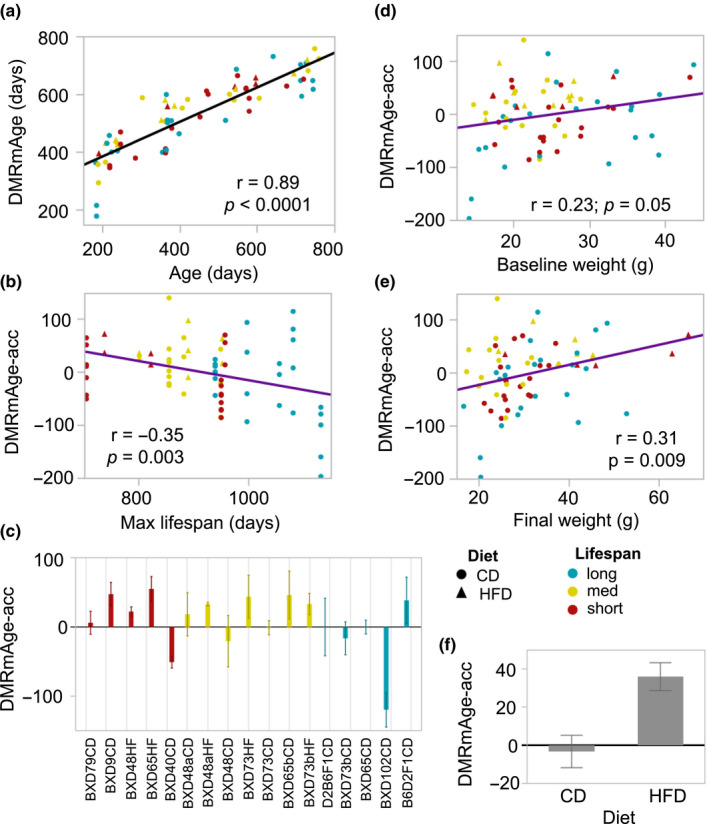
Age‐DMR‐based measure of epigenetic aging. (a) The epigenetic age of mice was estimated by taking the weighted averages of the 306 age‐DMRs. These age estimates, referred to as DMRmAge, have a strong positive correlation with the chronological age of mice (*n* = 69). (b) The age acceleration residuals (DMRmAge‐acc) derived from this clock have a significant negative correlation with the maximum life span data for the 17 strain‐by‐diet groups. (c) The bar plots show the mean DMRmAge‐acc values for each strain‐by‐diet group (error bars are standard error) with the graph ordered by increasing mean life span (*x*‐axis). The DMRmAge‐acc is positively correlated with both (d) body weight at young adulthood and (e) final body weight. (f) In the BXD strains with matched samples from both control diet (CD) and high‐fat diet (HFD), the DMRmAge‐acc is significantly higher in the HFD group compared to the CD group (–3.22 ± 36.99 in CD, 36.13 ± 27.32 in HFD, *p* = 0.002, *n* = 33).

We next evaluated whether we can derive a measure of differential rates of aging from the age‐DMRs that could be predictive of life span differences. We first summarized the age‐dependent changes by computing the weighted averages for the set of 306 age‐DMRs. The weighted averages, as expected, had a strong correlation with the chronological age of mice, and for a more direct comparison, the values were scaled to the age range for the 69 samples (Table S1). We refer to this DMR‐based age estimate as DMRmAge, and this showed a nearly linear correlation with chronological age at *r* = 0.88 (*p* < 0.0001, *n* = 69; Figure 5a). Age acceleration was derived as the residuals from the regression of DMRmAge on chronological age (Horvath, 2013; Thompson et al., 2018), with positive values indicating an older or accelerated biological age, and negative values indicating a decelerated biological age, and we refer to this as DMRmAge‐acc (Table S1).

The DMRmAge‐acc showed a significant negative correlation with strain maximum life span, indicating higher age acceleration in mice belonging to short‐lived strains (*r *= –0.35, *p* = 0.003, *n* = 69; Figure 5b). The correlation remained significant when the analysis was limited to only the CD mice (*r* = –0.30, *p* = 0.03, *n* = 55). There were notable strain differences in average DMRmAge‐acc (Figure 5c). We note that BXD65, which was the strain with the largest reduction in life span on HFD (Figure 1a), also showed a much higher age acceleration in the HFD group (mean DMRmAge‐acc = 54.94) compared to the CD group (mean DMRmAge‐acc = –0.17). The DMRmAge‐acc showed a significant positive correlation with both BW0 (*r* = 0.23, *p* = 0.05; Figure 5d) and final body weight of mice (*r* = 0.31, *p* = 0.009; Figure 5e) that suggests more accelerated aging with higher body weight. Limiting to only the CD mice (*n* = 55), the correlation with BW0 became slightly stronger (*r* = 0.29, *p* = 0.03), but the correlation with final weight became weaker (*r* = 0.23, *p* = 0.09, *n* = 14). We tested the effect of diet on the DMRmAge‐acc in the BXDs that had matched samples from both diets (*n* = 33), and this showed a significantly higher age acceleration in the HFD group (36.13 ± 27.32 in HFD, –3.22 ± 36.99 in CD, *p* = 0.002; Figure 5e). With the exception of one BXD73 sample that had spent 614 days on HFD, all the remaining HFD samples, including the ones that spent <100 days on HFD, showed positive DMRmAge‐acc values (Table S1).

We then treated the DNAmAge‐acc as the outcome variable, and used multivariable regression to evaluate the association with body weight (BW0 or final weight), diet, and the strain maximum life span. In the model with BW0 as an explanatory variable, the DNAmAge‐acc was significantly associated with life span (*F*_(1,65)_ = 6.3, *β* = –0.15, *p* = 0.01), BW0 (*F*_(1,65)_ = 6.2, *β* = 2.4, *p* = 0.02), and diet (*F*_(1,65)_ = 4.5, *p* = 0.04). In the model with final body weight, life span (*F*_(1,65)_ = 6.8, *β* = –0.16, *p* = 0.01) and body weight (*F*_(1,65)_ = 3.9, β = 1.5, *p* = 0.05), but not diet, were significant predictors. This suggests that the effect of diet on the clock is primarily mediated by the increase in final body weight.

To verify that these associations are robust, we repeated the EWAS using a randomly selected subset of 55 female methylomes (these are identified in Table S1). With the reduced sample size, 237 CpG regions were age‐DMRs at *p* ≤ 1.0 × 10^−4^. Using these age‐DMRs and respective regression coefficients from the subsample analysis, we re‐calculate the DMRmAge and DMRmAge‐acc (Table S1), and evaluated whether the age acceleration will show consistent associations with life span, body weight, and diet in the 55 subsamples, and also in the 14 excluded samples (the 3 male samples were assigned to this test set). The DMRmAge was significantly correlated with chronological age in both sets with *r* = 0.83 in the 55 subsamples, and *r* = 0.92 in the test set of 14 samples (Figure S3a). The DMRmAge‐acc defined from this continued to show inverse correlation with life span, and this was highly significant in the test set (Figure S3b). Notably, the two BXD102 (long‐lived strain) samples in the test set, including the male BXD102, had the most decelerated clocks among the 14 (Table S1). Both BW0 and the final body weight were positively correlated with the DMRmAge‐acc, although this did not reach statistical significance in either sets. Four HFD mice had been randomly assigned to the test set, and all four had positive DMRmAge‐acc. In the subsampled set, the HFD group had significantly higher age acceleration compared to the strain‐matched CD mice (60.49 ± 33.05 for HFD, 3.87 ± 55.40 for CD, *p* = 0.008, *n* = 25; Figure S3c). Similarly, HFD was associated with accelerated epigenetic aging in the strain‐matched samples in the test set (43.57 ± 19.83 for HFD, –12.02 ± 19.98 for CD, *p* = 0.008, *n* = 8; Figure S2c).

Taken together, the analysis demonstrates three points. First, that the age‐DMR‐based estimates of age acceleration are predictive of strain‐dependent differences in life span among female BXDs; second, that higher body weight, even before introduction to HFD, is associated with more accelerated aging; and third, HFD, which results in strain‐dependent increase in body weight, significantly accelerates aging, as measured by DMRmAge‐acc.

## 3 DISCUSSION

### 3.1 Genomic features of differentially methylated regions

In the present study, we parsed the variance in the methylome and defined site‐specific methylation differences that may be attributed to a strain‐level phenotype, median life span, and two individual‐level variables—age and BW0. For the age‐DMRs, the time‐dependent patterns were consistent with previous reports (Ciccarone, Tagliatesta, Caiafa, & Zampieri, 2018; Sziraki et al., 2018). As in Sziraki et al. (2018), methylation loss over time occurred mostly in regions with higher average DNAm, and methylation gains occurred mostly in regions with lower average DNAm. Since DNAm was quantified over 150 bp non‐overlapping bins, we were also able to relate the differential methylation patterns to the local CpG density. In particular, for the age‐hypermethylated DMRs, the increase in DNAm with aging had a strong positive correlation with CpG density. This too is consistent with reports that CpG dense regions—a feature of CpG islands, which typically remain unmethylated—are the sites that tend to gain methylation with age (Rakyan et al., 2010). The genes represented by the age‐DMRs included a few notable members such as *Cyp46a1* and *Abca7*, which are involved in cholesterol metabolism and implicated in Alzheimer's disease (Carter, 2007), and few members of the WNT signaling and mesenchyme developmental pathways (e.g., *Fzd1*, *Fzd8*, *Wnt5a*, *Jak3*,* Ptk7*, *Nrp2*). The current data replicated the CpG islands in *C1ql3* and *Ptk7*, which we previously reported as age‐hypermethylated sites in the BXD parental strains, B6 and D2 (Mozhui & Pandey, 2017). A region‐based functional annotation analysis revealed a significant enrichment in genes involved in cell polarity, an aspect of cells that is established during development through interaction with the WNT polarity signaling pathway, and which becomes dysregulated during aging (Berger, Wodarz, & Borchers, 2017; Budovsky, Fraifeld, & Aronov, 2011).

Body weight, even at young adulthood and before introduction to HFD, was significantly associated with the global methylome patterns and was also predictive of strain longevity. We therefore included BW0 as a predictor variable in the multiple regression model. The BW0‐DMRs included a few genes that have been previously associated with body weight in human GWAS at *p* < 5 × 10^−8^(Buniello et al., 2019). This included an intron DMR in the fat mass and obesity associated *Fto* gene, which plays a key role in energy homeostasis, and consistently shown to influence body weight in humans (Zhou, Simmons, Lai, Hambly, & McLachlan, 2017). The BW0‐DMRs were mostly located within genes, particularly introns, and a striking aspect of the BW0‐DMRs was that 75% were negatively associated with BW0. For the BW0‐DMRs that were linked to the corresponding transcript, the overall pattern indicated a negative correlation between DNAm and gene expression. This means that while DNAm at these sites were, in general, inversely correlated with body weight, the transcript levels of the corresponding genes generally had higher expression in mice that were heavier.

For the longevity trait, only 124 CpG regions were significant at the suggestive threshold. A point of distinction for the LS‐DMRs is that, while the age‐ and BW0‐DMRs were related to individual‐level variables, the LS‐DMRs were related to life expectancy based on the strain and the diet. This indirect association with the phenotype may explain why only few DMRs were uncovered by the current mixed model. Given this small number of genes, it is particularly striking that the region‐based annotation revealed that genes that cause premature death in single knockout mice are the most enriched gene set among the LS‐DMRs (phenotype ID MP:0002083, defined as “death after weaning, but before the normal life span” in http://www.informatics.jax.org). Among the LS‐DMRs, both *Tert* and *Igfbp2* have also been linked to aging and longevity by human GWAS (Lu et al., 2018; Teumer et al., 2016).

### 3.2 Building clocks from age‐dependent CpG regions

Several different versions of the DNAmAge estimator are now available for both mice and humans (Hannum et al., 2013; Horvath, 2013; Petkovich et al., 2017; Stubbs et al., 2017; Thompson et al., 2018; Wang et al., 2017). The standard protocol for developing DNAmAge clocks starts by applying a regression algorithm in a training dataset, followed by age estimation in validation cohorts to gauge the accuracy of the clock. Our goal in this study was not to develop another DNAmAge clock. Given the sample size of the present study, that would not have been a feasible pursuit. Instead, our goal was to test whether the age‐dependent CpG regions could provide an estimate of epigenetic aging that could discern life span differences between the mouse strains, and that could be related to body weight and diet. For this, we simply summarized the age‐DMRs by computing the weighted averages for each sample. The weighted averages, unsurprisingly, correlated strongly with the chronological age of mice. More importantly, the age acceleration derived from the age‐DMR‐based clock was (a) inversely correlated with the strain life span phenotype, (b) was significantly more accelerated in the HFD group, and (c) was positively correlated with body weight. This conveys that the age‐DMRs can estimate genetically modulated differences in rates of biological aging and life span, and is a modifiable outcome. Furthermore, the results highlight the interdependence between body weight, diet, and health and aging, and our observations agree with the well‐known influence of body mass on longevity, and the more favorable health profile associated with lower body mass within a species (Bartke, 2012).

The strain with the most decelerated clock, and presumably slowest rate of biological aging, was BXD102 on CD, which is among the longest‐lived BXD strain we had in the study. The DMRmAge‐acc for this strain ranged from –78 to –196 days for the females and –66 days for the one male sample. The subsamples analysis in the 55 female cases recapitulated this decelerated clock in BXD102. However, we note that there were a few mismatches between strain life span classification and the DMRmAge‐acc. BXD40 on CD, although classified as short‐lived, was the only short‐lived strain with a mean negative DMRmAge‐acc. B6D2F1, on the other hand, although classified as long‐lived, was the only long‐lived group with mean positive DMRmAge‐acc. Unlike the inbred BXDs, the heterozygous F1s have hybrid vigor in both body weight and life span, and this likely explains the inconsistency.

### 3.3 Technical considerations and limitations

Before concluding, we should address a few caveats. The sequence alignment was done to the mm10 B6 reference genome, which means that for regions with genetic variants segregating in the BXDs, the sequence differences could compromise alignment. Chronological age, at least in the *biospecimen cohort*, is independent of genetic background, and the age‐DMRs are expected to be less susceptible to the confounding effect of DNA sequence variants. For body weight and life span, the interpretation is complicated by the fact that both phenotypes are closely linked to genotype, and the DMRs may reflect true differences in DNAm levels, or differential quantification due to sequence effects. To partly control for this, we used a mixed model that fitted each strain‐diet group as a random intercept. For the age‐DMRs, 17% of the CpG regions contained sequence variants, and this is similar to the 17% of variant containing bins in the background set of 368,300 regions. The BW0‐DMRs and LS‐DMRs were only slightly enriched in variant containing bins, and for the most part, the CpG regions were devoid of sequence differences.

To our knowledge, almost all existing DNAm clocks have relied on bisulfite‐based assays, which have the advantage of providing single CpG resolution (Hannum et al., 2013; Horvath, 2013; Levine et al., 2018; Petkovich et al., 2017; Stubbs et al., 2017; Thompson et al., 2018; Wang et al., 2017). For the mouse model, the reduced representation data (RRBS) generated by Petkovich et al. (2017) from ~141 B6 mice have been used in different studies to define tens of thousands of age‐dependent CpG sites (over 43,000 CpGs in Lowe et al. (2018), and over 146,000 in Sziraki et al. (2018)). Compared to these, the present work identified only 306 age‐DMRs at the suggestive threshold that covered 2691 CpG sites. This is still a relatively modest number of age‐dependent CpGs, and this is likely due to the small sample size and the genotype heterogeneity of the present cohort. Another contributing factor may be the methylome assay we used, as MBD‐seq provides lower quantitative sensitivity (Gujar, Liang, Wong, & Mozhui, 2018). Nonetheless, MBD‐sequencing still delivers highly sensitive and replicable quantification of genome‐wide methylation (Aberg et al., 2018). Using alternate assay methods also demonstrates that the DNAm age estimators are robust to the techniques used to quantify DNAm.

We note that the present study is specific to females. We included a few male cases in the methylome assay, and the age‐DMR‐based clock closely estimated the chronological ages of the three males as well. Longevity is reported to be highly strain‐dependent in the BXD panel, with significant correlation in median life span between male and female BXDs, but also with notable sex differences in the underlying genetics (Lang et al., 2010). The decelerated DMRmAge‐acc in the one male BXD102 also suggests strain similarity in epigenetic aging rate between a male and the females of a long‐lived strain. However, with such a limited sampling of male cases, no conclusion can be drawn, and their inclusion may have added more heterogeneity than information. However, excluding the male cases, as was done for the subset analysis in the 55 female samples, did not alter the main findings of the study.

## 4 EXPERIMENTAL PROCEDURES

### 4.1 Statistical analyses

Descriptions of animal protocols, sample processing for MBD‐seq, sequence alignment, initial bioinformatics, data filtering, and quality checks are provided in Appendix S1 and Figure S1. Additional description of statistics and transcriptome analysis are also described in Appendix S1.

For the EWAS to detect DMRs, we applied the following model using the lme4 R package (v4_1.1‐21) (Bates, Mächler, Bolker, & Walker, 2015): lmer(logRPKM ~age + BW0 + medianLifeSpan + (1|StrainDiet)). This was first applied to the full set of 69 samples. Manhattan plots were generated using the qqman R package (Turner, 2018). For the DMRs, we evaluated relative enrichment in genomic features (i.e., introns, exons, and CpG islands) compared to the background set using the hypergeometric test in R (R codes in Table S3). Following that, we carried out functional annotation and enrichment analysis against the whole background genome using the GREAT application (version 4.0.4) (McLean et al., 2010). Enrichment was calculated using both a binomial test and a hypergeometric test, and we report only categories that were significant by both methods (FDR < 0.05). To search for human GWAS hits, we referred to the GWAS catalog (Buniello et al., 2019), and searched for the terms “body weight”, “aging”, and “longevity”. For variants with reported association *p* ≤ 5 × 10^−8^, the mapped human gene symbols were then matched to the corresponding mouse genes associated with the DMR.

### 4.2 Estimating epigenetic age and age acceleration

To estimate epigenetic age, we computed the weighted averages with each of the 306 age‐DMRs weighed by the respective age regression coefficient. The weighted averages were then scaled to the age range in the 69 samples using the following formula: DMRmAge = [(((weighted.average − min.weighted.average) × age.range)/weighted.average.range) + min.age], where min.weighted.average and weighted.average.range are the minimum value and range for the weighted averages in the 69 BXD samples, and age.range = 578 days is the range of chronological age in the 69 BXDs, and min.age = 181 days is the minimum age for the 69 BXDs. As recommended in Thompson et al. 2018 (Thompson et al., 2018), the “age acceleration” was computed as the residuals after fitting the predicted age to chronological ages: residuals(lm(DMRmAge ~Age)).

The DMR EWAS was repeated using the same mixed model (lmer(logRPKM ~age + BW0 + medianLifeSpan + (1|StrainDiet))) in a randomly selected subset of 55 female samples. Random subsampling was done using the R function, sample() with *n* = 55 and replace = FALSE, after excluding the 3 males. From this, age‐DMRs (age‐associated at *p* ≤ 1.0 × 10^−4^, 237 CpG regions) and the respective regression coefficients were then used to re‐compute the DMRmAge using the same weighted averaging method described above. We then used the 14 samples excluded from the EWAS as a test set to examine the association of DMRmAge‐acc with life span, body weight, and diet.

## 5 CONCLUSION

Our results demonstrate that the epigenetic clock defined from age‐DMRs is sensitive to subtle differences in natural life span among female mice that arise from common genetic variants, and is modifiable by environmental interventions, such as diet. The intercorrelations between epigenetic aging, body weight, and longevity also provide evidence that the methylome could provide a mechanistic link between the well‐known effect of body mass on aging and life span.

## CONFLICT OF INTEREST

No competing interests.

## AUTHOR CONTRIBUTIONS

KM supervised the study, contributed to conception of study design and analysis, and wrote the manuscript. RWW is the principal investigator of the BXD Aging Colony and contributed to the conception of the aging study, and provided access to the biorepository resource. JVSS, AHBH, and EGW contributed to the laboratory work, and JVSS performed the primary bioinformatics and initial data processing. DA and SR contributed to analysis and data acquisition. All authors contributed to and approved the final version of the manuscript.

## ETHICAL APPROVAL

All animal procedures were in accordance to protocol approved by the Institutional Animal Care and Use Committee (IACUC) at the University of Tennessee Health Sciencne Center.

## Supporting information

Fig S1‐S3

Appendix S1

Tables S1‐S11

## Data Availability

The normalized MBD‐seq data for the 368,300 CpG bins (including chromosomal coordinates, region annotations, CpG density, and variant counts) are available from the NCBI NIH Gene Expression Omnibus. The full raw fastq files are available from the NCBI NIH Sequence Repository Archive. GEO accession ID is GSE137277, and the linked SRA accession ID is SRP221380.

## References

[acel13207-bib-0001] Aberg, K. A. , Chan, R. F. , Xie, L. , Shabalin, A. A. , & van den Oord, E. (2018). Methyl‐CpG‐binding domain sequencing: MBD‐seq. Methods in Molecular Biology, 1708, 171–189. 29224145 10.1007/978-1-4939-7481-8_10

[acel13207-bib-0002] Ashbrook, D. G. , Arends, D. , Prins, P. , Mulligan, M. K. , Roy, S. , Williams, E. G. , … Willaims, R. W. (2019). The expanded BXD family of mice: A cohort for experimental systems genetics and precision medicine. bioRxiv, 672097.

[acel13207-bib-0003] Bartke, A. (2012). Healthy aging: is smaller better? ‐ A mini‐review. Gerontology, 58(4), 337–343. 22261798 10.1159/000335166 PMC3893695

[acel13207-bib-0004] Bates, D. , Mächler, M. , Bolker, B. , & Walker, S. (2015). Fitting Linear Mixed‐Effects Models Using lme4. Journal of Statistical Software, 67 http://CRANR‐projectorg/package=lme4

[acel13207-bib-0005] Berger, H. , Wodarz, A. , & Borchers, A. (2017). PTK7 Faces the Wnt in development and disease. Frontiers in Cell and Developmental Biology, 5, 31. 28424771 10.3389/fcell.2017.00031 PMC5380734

[acel13207-bib-0006] Breitling, L. P. , Saum, K. U. , Perna, L. , Schottker, B. , Holleczek, B. , & Brenner, H. (2016). Frailty is associated with the epigenetic clock but not with telomere length in a German cohort. Clinical Epigenetics, 8, 21. 26925173 10.1186/s13148-016-0186-5 PMC4768341

[acel13207-bib-0007] Budovsky, A. , Fraifeld, V. E. , & Aronov, S. (2011). Linking cell polarity, aging and rejuvenation. Biogerontology, 12(2), 167–175. 20978937 10.1007/s10522-010-9305-4

[acel13207-bib-0008] Buniello, A. , MacArthur, J. A. L. , Cerezo, M. , Harris, L. W. , Hayhurst, J. , Malangone, C. , … Suveges, D. (2019). The NHGRI‐EBI GWAS Catalog of published genome‐wide association studies, targeted arrays and summary statistics 2019. Nucleic Acids Research, 47(D1), D1005–D1012. 30445434 10.1093/nar/gky1120 PMC6323933

[acel13207-bib-0009] Carter, C. J. (2007). Convergence of genes implicated in Alzheimer's disease on the cerebral cholesterol shuttle: APP, cholesterol, lipoproteins, and atherosclerosis. Neurochemistry International, 50(1), 12–38. 16973241 10.1016/j.neuint.2006.07.007

[acel13207-bib-0010] Chen, B. H. , Marioni, R. E. , Colicino, E. , Peters, M. J. , Ward‐Caviness, C. K. , Tsai, P. C. , … Bressler, J. (2016). DNA methylation‐based measures of biological age: meta‐analysis predicting time to death. Aging, 8(9), 1844–1865. 27690265 10.18632/aging.101020 PMC5076441

[acel13207-bib-0011] Ciccarone, F. , Tagliatesta, S. , Caiafa, P. , & Zampieri, M. (2018). DNA methylation dynamics in aging: How far are we from understanding the mechanisms? Mechanisms of Ageing and Development, 174, 3–17. 29268958 10.1016/j.mad.2017.12.002

[acel13207-bib-0012] Cole, J. J. , Robertson, N. A. , Rather, M. I. , Thomson, J. P. , McBryan, T. , Sproul, D. , … Meehan, R. R. (2017). Diverse interventions that extend mouse lifespan suppress shared age‐associated epigenetic changes at critical gene regulatory regions. Genome Biology, 18, 1–16. 28351383 10.1186/s13059-017-1185-3 PMC5370462

[acel13207-bib-0013] de Haan, G. , Gelman, R. , Watson, A. , Yunis, E. , & Van Zant, G. (1998). A putative gene causes variability in lifespan among genotypically identical mice. Nature Genetics, 19(2), 114–116. 9620762 10.1038/465

[acel13207-bib-0014] De Haan, G. , & Van Zant, G. (1999). Genetic analysis of hemopoietic cell cycling in mice suggests its involvement in organismal life span. FASEB Journal: Official Publication of the Federation of American Societies for Experimental Biology, 13(6), 707–713. 10094931 10.1096/fasebj.13.6.707

[acel13207-bib-0015] Gelman, R. , Watson, A. , Bronson, R. , & Yunis, E. (1988). Murine chromosomal regions correlated with longevity. Genetics, 118(4), 693–704. 3163317 10.1093/genetics/118.4.693 PMC1203324

[acel13207-bib-0016] Gujar, H. , Liang, J. W. , Wong, N. C. , & Mozhui, K. (2018). Profiling DNA methylation differences between inbred mouse strains on the Illumina Human Infinium MethylationEPIC microarray. PLoS One, 13(3), e0193496. 29529061 10.1371/journal.pone.0193496 PMC5846735

[acel13207-bib-0017] Hannum, G. , Guinney, J. , Zhao, L. , Zhang, L. , Hughes, G. , Sadda, S. , … Deconde, R. (2013). Genome‐wide methylation profiles reveal quantitative views of human aging rates. Molecular Cell, 49(2), 359–367. 23177740 10.1016/j.molcel.2012.10.016 PMC3780611

[acel13207-bib-0018] Horvath, S. (2013). DNA methylation age of human tissues and cell types. Genome Biology, 14(10), R115. 24138928 10.1186/gb-2013-14-10-r115 PMC4015143

[acel13207-bib-0019] Hsu, H. C. , Li, L. , Zhang, H. G. , & Mountz, J. D. (2005). Genetic regulation of thymic involution. Mechanisms of Ageing and Development, 126(1), 87–97. 15610766 10.1016/j.mad.2004.09.016

[acel13207-bib-0020] Lang, D. H. , Gerhard, G. S. , Griffith, J. W. , Vogler, G. P. , Vandenbergh, D. J. , Blizard, D. A. , & McClearn, G. E. (2010). Quantitative trait loci (QTL) analysis of longevity in C57BL/6J by DBA/2J (BXD) recombinant inbred mice. Aging Clinical and Experimental Research, 22(1), 8–19. 20305363 10.1007/BF03324809

[acel13207-bib-0021] Levine, M. E. , Lu, A. T. , Quach, A. , Chen, B. H. , Assimes, T. L. , Bandinelli, S. , … Whitsel, E. A. (2018). An epigenetic biomarker of aging for lifespan and healthspan. Aging, 10(4), 573–591. 29676998 10.18632/aging.101414 PMC5940111

[acel13207-bib-0022] Lowe, R. , Barton, C. , Jenkins, C. A. , Ernst, C. , Forman, O. , Fernandez‐Twinn, D. S. , … Rakyan, V. K. (2018). Ageing‐associated DNA methylation dynamics are a molecular readout of lifespan variation among mammalian species. Genome Biology, 19(1), 22. 29452591 10.1186/s13059-018-1397-1 PMC5815211

[acel13207-bib-0023] Lu, A. T. , Quach, A. , Wilson, J. G. , Reiner, A. P. , Aviv, A. , Raj, K. , … Whitsel, E. A. (2019). DNA methylation GrimAge strongly predicts lifespan and healthspan. Aging, 11(2), 303–327. 30669119 10.18632/aging.101684 PMC6366976

[acel13207-bib-0024] Lu, A. T. , Xue, L. , Salfati, E. L. , Chen, B. H. , Ferrucci, L. , Levy, D. , … Horvath, S. (2018). GWAS of epigenetic aging rates in blood reveals a critical role for TERT. Nature Communications, 9(1), 387. 10.1038/s41467-017-02697-5 PMC5786029 29374233

[acel13207-bib-0025] Marioni, R. E. , Harris, S. E. , Shah, S. , McRae, A. F. , von Zglinicki, T. , Martin‐Ruiz, C. , … Deary, I. J. (2018). The epigenetic clock and telomere length are independently associated with chronological age and mortality. International Journal of Epidemiology, 47(1), 356. 29190382 10.1093/ije/dyx233 PMC5837660

[acel13207-bib-0026] Marioni, R. E. , Shah, S. , McRae, A. F. , Chen, B. H. , Colicino, E. , Harris, S. E. , … Pattie, A. (2015). DNA methylation age of blood predicts all‐cause mortality in later life. Genome Biology, 16(1), 25. 25633388 10.1186/s13059-015-0584-6 PMC4350614

[acel13207-bib-0027] Marioni, R. E. , Shah, S. , McRae, A. F. , Ritchie, S. J. , Muniz‐Terrera, G. , Harris, S. E. , … Corley, J. (2015). The epigenetic clock is correlated with physical and cognitive fitness in the Lothian Birth Cohort 1936. International Journal of Epidemiology, 44(4), 1388–1396. 25617346 10.1093/ije/dyu277 PMC4588858

[acel13207-bib-0028] McLean, C. Y. , Bristor, D. , Hiller, M. , Clarke, S. L. , Schaar, B. T. , Lowe, C. B. , … Bejerano, G. (2010). GREAT improves functional interpretation of cis‐regulatory regions. Nature Biotechnology, 28(5), 495–501. 10.1038/nbt.1630 PMC4840234 20436461

[acel13207-bib-0029] Metrustry, S. J. , Edwards, M. H. , Medland, S. E. , Holloway, J. W. , Montgomery, G. W. , Martin, N. G. , … Valdes, A. M. (2014). Variants close to NTRK2 gene are associated with birth weight in female twins. Twin Research and Human Genetics, 17(4), 254–261. 24950379 10.1017/thg.2014.34

[acel13207-bib-0030] Mozhui, K. , & Pandey, A. K. (2017). Conserved effect of aging on DNA methylation and association with EZH2 polycomb protein in mice and humans. Mechanisms of Ageing and Development, 162, 27–37. 28249716 10.1016/j.mad.2017.02.006 PMC5411177

[acel13207-bib-0031] Petkovich, D. A. , Podolskiy, D. I. , Lobanov, A. V. , Lee, S. G. , Miller, R. A. , & Gladyshev, V. N. (2017). Using DNA methylation profiling to evaluate biological age and longevity interventions. Cell Metabolism, 25(4), 954–960e956. 28380383 10.1016/j.cmet.2017.03.016 PMC5578459

[acel13207-bib-0032] Rakyan, V. K. , Down, T. A. , Maslau, S. , Andrew, T. , Yang, T. P. , Beyan, H. , … Spector, T. D. (2010). Human aging‐associated DNA hypermethylation occurs preferentially at bivalent chromatin domains. Genome Research, 20(4), 434–439. 20219945 10.1101/gr.103101.109 PMC2847746

[acel13207-bib-0033] Roy, S. , Sleiman, M. B. , Jha, P. , Williams, E. G. , Ingels, J. F. , Chapman, C. J. , … Williams, R. W. (2019). Gene‐by‐environmental modulation of longevity and weight gain in the murine BXD family. bioRxiv, 776559.

[acel13207-bib-0034] Sloane, L. B. , Stout, J. T. , Vandenbergh, D. J. , Vogler, G. P. , Gerhard, G. S. , & McClearn, G. E. (2011). Quantitative trait loci analysis of tail tendon break time in mice of C57BL/6J and DBA/2J lineage. The Journals of Gerontology Series A, Biological Sciences and Medical Sciences, 66(2), 170–178. 10.1093/gerona/glq169 PMC3021371 21047976

[acel13207-bib-0035] Stubbs, T. M. , Bonder, M. J. , Stark, A. K. , Krueger, F. , von Meyenn, F. , Stegle, O. , & Reik, W. (2017). Multi‐tissue DNA methylation age predictor in mouse. Genome Biology, 18(1), 68. 28399939 10.1186/s13059-017-1203-5 PMC5389178

[acel13207-bib-0036] Sziraki, A. , Tyshkovskiy, A. , & Gladyshev, V. N. (2018). Global remodeling of the mouse DNA methylome during aging and in response to calorie restriction. Aging Cell, 17(3), e12738. 29575528 10.1111/acel.12738 PMC5946071

[acel13207-bib-0037] Teumer, A. , Qi, Q. , Nethander, M. , Aschard, H. , Bandinelli, S. , Beekman, M. , … Kaplan, R. C. (2016). Genomewide meta‐analysis identifies loci associated with IGF‐I and IGFBP‐3 levels with impact on age‐related traits. Aging Cell, 15(5), 811–824. 27329260 10.1111/acel.12490 PMC5013013

[acel13207-bib-0038] Thompson, M. J. , Chwialkowska, K. , Rubbi, L. , Lusis, A. J. , Davis, R. C. , Srivastava, A. , … Pellegrini, M. (2018). A multi‐tissue full lifespan epigenetic clock for mice. Aging, 10(10), 2832–2854. 30348905 10.18632/aging.101590 PMC6224226

[acel13207-bib-0039] Thorleifsson, G. , Walters, G. B. , Gudbjartsson, D. F. , Steinthorsdottir, V. , Sulem, P. , Helgadottir, A. , … Stefansson, K. (2009). Genome‐wide association yields new sequence variants at seven loci that associate with measures of obesity. Nature Genetics, 41(1), 18–24. 19079260 10.1038/ng.274

[acel13207-bib-0040] Turner, S. (2018). qqman: An R package for visualizing GWAS results using Q‐Q and manhattan plots. Journal of Open Source Software, 3(25), 731.

[acel13207-bib-0041] Wang, T. , Tsui, B. , Kreisberg, J. F. , Robertson, N. A. , Gross, A. M. , Yu, M. K. , … Ideker, T. (2017). Epigenetic aging signatures in mice livers are slowed by dwarfism, calorie restriction and rapamycin treatment. Genome Biology, 18(1), 57. 28351423 10.1186/s13059-017-1186-2 PMC5371228

[acel13207-bib-0042] Weedon, M. N. , Lettre, G. , Freathy, R. M. , Lindgren, C. M. , Voight, B. F. , Perry, J. R. B. , … Frayling, T. M. (2007). A common variant of HMGA2 is associated with adult and childhood height in the general population. Nature Genetics, 39(10), 1245–1250. 17767157 10.1038/ng2121 PMC3086278

[acel13207-bib-0043] Yuan, R. , Peters, L. L. , & Paigen, B. (2011). Mice as a mammalian model for research on the genetics of aging. ILAR Journal/National Research Council, Institute of Laboratory Animal Resources, 52(1), 4–15. 10.1093/ilar.52.1.4 PMC3074346 21411853

[acel13207-bib-0044] Yuan, R. , Tsaih, S. W. , Petkova, S. B. , De Evsikova, C. M. , Xing, S. , Marion, M. A. , … Rosen, C. J. (2009). Aging in inbred strains of mice: study design and interim report on median lifespans and circulating IGF1 levels. Aging Cell, 8(3), 277–287. 19627267 10.1111/j.1474-9726.2009.00478.x PMC2768517

[acel13207-bib-0045] Zhou, Y. , Simmons, D. , Lai, D. , Hambly, B. D. , & McLachlan, C. S. (2017). rs9939609 FTO genotype associations with FTO methylation level influences body mass and telomere length in an Australian rural population. International Journal of Obesity, 41(9), 1427–1433. 28559540 10.1038/ijo.2017.127

