# Corrigendum: Body weight and high‐fat diet are associated with epigenetic aging in female members of the BXD murine family

**DOI:** 10.1111/acel.13473

**Published:** 2021-09-15

Jose Vladimir Sandoval‐Sierra, Alexandra H. B. Helbing, Evan G. Williams, David G. Ashbrook, Suheeta Roy, Robert W. Williams, Khyobeni Mozhui, *Aging Cell*. 2020;19:e13207. https://doi.org/10.1111/acel.13207

First published: 12 August 2020.

In Sandoval‐Sierra et al. (2020), the authors noticed an error in Figure 1d. The x‐axis unit should be “days” and not “years.” The revised figure is shown below.

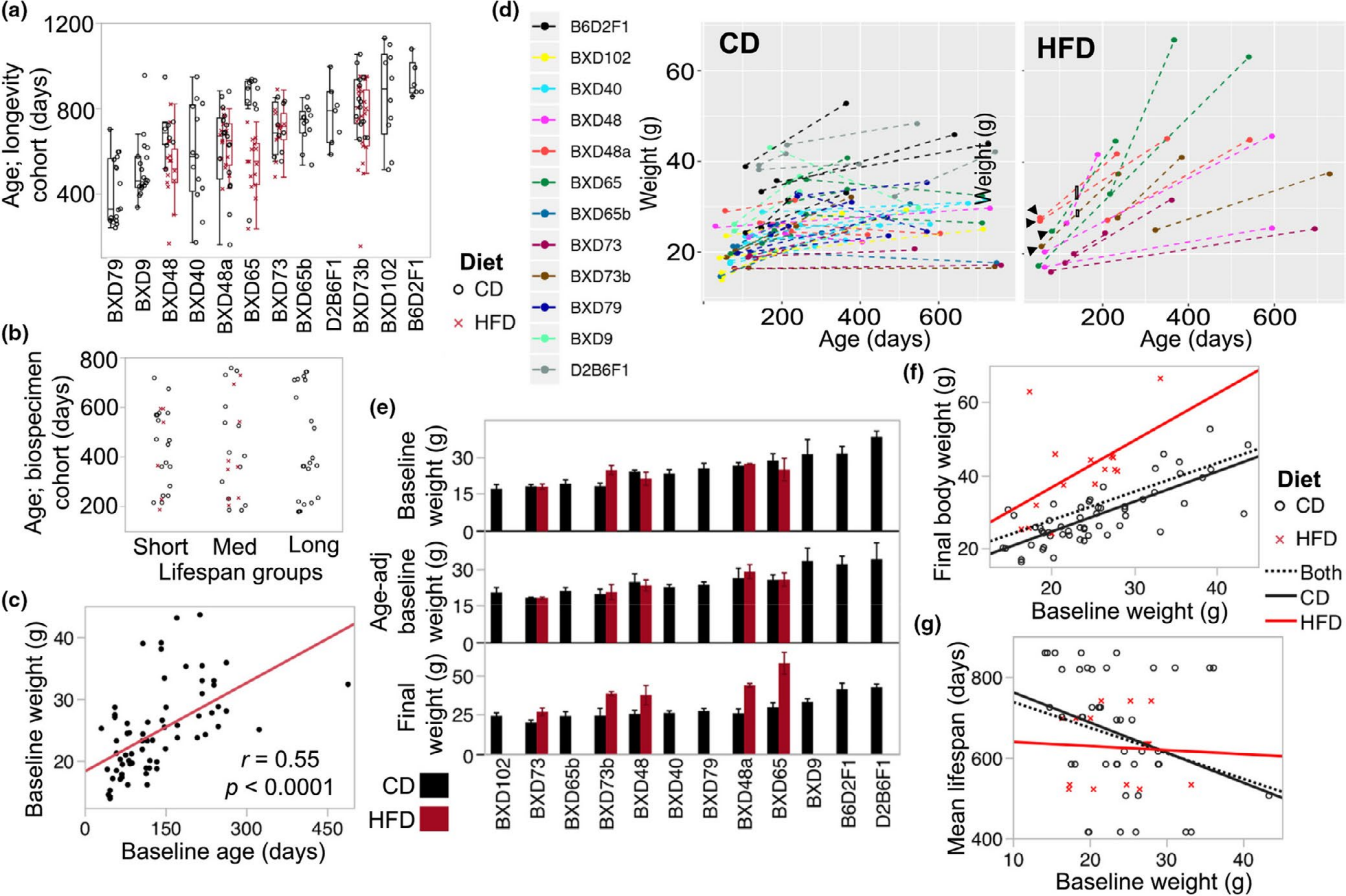

The authors apologize for the error.
