# Supplementary material for: Body weight and high‐fat diet are associated with epigenetic aging in female members of the BXD murine family
Source: Aging Cell. 2020 Aug 12;19(9):e13207. doi: 10.1111/acel.13207 (PMC7511861; doi:10.1111/acel.13207)
Supplement: Supplementary file 1 — Fig S1‐S3 [file ACEL-19-e13207-s001.docx]

**Supplementary Figures**

**Fig S1. Unsupervised hierarchical cluster and density plots**

**(a)** The dendrogram plotted using the genome-wide data shows that samples cluster by strain identity rather than age or diet. **(b)** Density plots of logRPKM values for the 368,300 CpGs bins show a consistent distribution. Methylation scores average is at 3.8 ± 0.72 logRPKM.

**Fig S2. Genome-wide mean methylation and correlations with principal components**

For each individual mouse, the overall mean methylation and within-individual variance was calculated for **(a)** 167,769 CpG regions in intergenic sites, and **(b)** 200,531 genic CpG regions located within annotated genes. The intergenic regions have wide variation between strains and the F1 hybrids have the highest mean methylation and lowest variance. The genic CpG regions are more consistent across strains. Mean methylation is inversely correlated with variance, and this is particularly pronounced for the intergenic CpG regions. Average methylation at intergenic regions (x-axis) is correlated with PC1 **(c)**, and PC3 **(d)**, and average methylation at genic regions is correlated with PC4 **(e)**.

**Fig. S3. Age-DMR based measure of epigenetic aging in subsamples and test sets**

**(a)** The epigenetic age, DMRmAge, was estimated using 237 age-dependent differentially methylated CpG regions (age-DMRs) defined from a subsampled set of 55 female methylomes. The DMRmAge has a strong positive correlation with the chronological age of mice in the 55 subsamples (left panel). The 14 samples excluded from the age-DMR definition included the three male cases (open circles), and these samples served as a test set, and the DMRmAge predicts chronological age in the test set as well (right panel). **(b)** The age acceleration residuals (DMRmAge-acc) derived from this clock has a significant negative correlation with the maximum lifespan in both the subsamples, and the test set, but is significant only in the test set. **(c)** In the BXD strains with matched samples from both control diet (CD) and high fat diet (HFD), the DMRmAge-acc is significantly higher in the HFD group compared to the CD group (60.49 ± 33.05 for HFD, 3.87 ± 55.40 for CD, *p* = 0.008, *n* = 25). HFD also accelerated DMRmAge in the strain-matched samples in the test set (43.57 ± 19.83 for HFD, –12.02 ± 19.98 for CD, *p* = 0.008, *n* = 8).
