# Supplementary material for: Body weight and high‐fat diet are associated with epigenetic aging in female members of the BXD murine family
Source: Aging Cell. 2020 Aug 12;19(9):e13207. doi: 10.1111/acel.13207 (PMC7511861; doi:10.1111/acel.13207)
Supplement: Supplementary file 2 — Appendix S1 [file ACEL-19-e13207-s002.docx]

**Appendix S1**

**Animals**

The present study utilizes biobanked liver samples collected from mice that were housed in a specific pathogen-free (SPF) facility at the University of Tennessee Health Science Center (UTHSC). Mice were kept at 20–24 °C on a 12-hour light cycle. The control diet (CD) animals were given *ad libitum* access to water and standard chow (18% calories from fat). Upon initial weighing, mice assigned to high fat diet (HFD) were given *ad libitum* access to water and high fat chow (60.3% calories from fat). Details of the diet are provide in Roy et al., ^1^. Mice that were selected for tissue collection were periodically euthanized at ages that ranged from 6 months to >24 months. Mice were first deeply anesthetized with avertin (0.02 ml per g of body weight), and were then perfused with ice cold phosphate-buffered saline. Liver was removed, weighed, and immediately frozen in liquid nitrogen, and then stored in –80 °C. All animal procedures were in accordance to protocol approved by the Institutional Animal Care and Use Committee (IACUC) at the University of Tennessee Health Science Center.

**Statistics**

Correlations between continuous variables (e.g., age, weight measurements, strain lifespan data, principal components, methylation averages) were done using Pearson correlation, and t-test was used to evaluate the effect of diet as a categorical predictor. For age-corrected baseline body weight (BW0), residual values were derived by regressing BW0 on baseline age, and values were then scaled to the original BW0 range. Multi-variable analyses were based on linear regression.

**Sample preparation**

DNA was extracted using the DNeasy Blood & Tissue Kit from Qiagen. Nucleic acid purity was inspected by using a NanoDrop spectrophotometer, and quantified using a Qubit fluorometer dsDNA BR Assay. Affinity-based enrichment was carried out using the MethylMiner DNA enrichment kit from ThermoFisher Scientific according to the manufacturer’s standard protocol. In brief, 1 μg of DNA in 110 μl of low TE buffer was fragmented to ~150 bp using a Covaris S2 ultrasonicator. Sonication settings were: cycle/burst of 1 for 10 cycles of 60 s, duty cycle of 10%, intensity of 5.0. DNA fragment size and quality were assessed using the Agilent Bioanalyzer 2100. Following MBD capture reactions, DNA was eluted in a single step using high salt (2000 mM NaCl) elution buffer, and re-concentrated by ethanol precipitation. The final concentration of methylated-CpG enriched DNA ranged from 1.12 to 5.43 ηg per μl (2.46 ± 0.94). Sequencing was done to a depth of approximately 50 million reads per sample (150 bp paired-end) on the Illumina HiSeq 4000, and carried out by Novogene (<https://en.novogene.com>).

**Read alignment and initial data processing**

FASTQ files were first inspected with the FastQC tool (v.0.11.8) ^2^, and alignment was done to the mouse reference genome (mm10/GRCm38) using Bowtie2 (v.2.3.4.3) ^3^. Alignment quality was evaluated with SAMtools (v.1.9) ^4^, and SAMstat v.1.5.1^5^. Potential PCR duplicates and reads with mapping quality less than 10 were removed. The bam files were loaded to the MEDIPS R package (v.1.36.0) ^6^ for additional quality checks and assessment of read coverage. Saturation analysis (MEDIPS.saturation) showed that all 70 libraries had sufficient read coverage, and pair-wise correlations (MEDIPS.correlation) showed high consistency between samples (Pearson *r* > 0.90 for all pairs). Given the MBD enrichment, all the samples were enriched for CpGs (mean CG enrichment score of 2.30 ± 0.19; using the MEDIPS MEDIPS.CpGenrich function), and on average, 51% of CpGs in the reference genome was covered by at least one mapped read, with 28% of CpGs covered by > 5 mapped reads. To quantify DNAm, the mouse genome was divided in 150 bp non-overlapping windows and reads were counted for each bin with normalization to the local CpG density (coupling factor or CF) using the function MEDIPS.meth and the following parameters: ws =150, extend = 150, uniq = 1, shift=0. Read counts were then filtered to retain only 150 bp bins that had sufficient coverage for reliable quantification and statistical analyses. First, bins with no CpGs (CF = 0) and mean read counts ≤ 1 were excluded, resulting in 4,286,826 bins. The Y chromosome was also excluded. The filtered data was loaded to the EdgeR R package (v3.24) ^7^ and further filtered on the basis of counts per million (CPM) to retain only reads with more than 1 CPM in 2 or more libraries. This resulted in 368,300 CpG regions with sufficient coverage across the libraries, and these were normalized using the calcNormFactors function. RPKM values were then extracted using the parameters gene.length = 150, log = TRUE. The compendium of SNPs and small insertions/deletions segregating in the BXDs have been catalogued for the BXDs ^8^, and we used this information to count the number of variants in each of the 368,300 150 bp bins.

These CpG regions were annotated for genomic features using the HOMER program (v4.10) ^9^. The CpG regions were then divided into bins that occurred within annotated genes (genic set, 200,531 bins), and those that were in intergenic regions (167,769 bins). For each sample, the overall average methylation and variance for these genic and intergenic sets were computed. The intercorrelations between the large-scale methylome features, body weight measures, and strain-level lifespan phenotype were examined using Pearson correlations.

PCA and hierarchical clustering was done in R for this full set of bins (**Fig S1a**). These detected no outlier samples and the DNAm profiles were consistent across the samples (**Fig S1b**), and averaged at 3.8 ± 0.72 logRPKM.

**Transcriptomes analyses**

We used liver gene expression data that are available from GeneNetwork (<http://genenetwork.org>; data accession ID GN877, UTHSC BXD Harvested Liver RNA-Seq (Oct19)). While this is a bigger cohort, we limited the analysis to only 52 mice that had matched MBD-seq in the present study. Gene expression was log_2_ transformed transcripts per million (TPM). For the age-, BW0-, and LS-DMRs, the cognate genes were defined as the gene in which the DMR is located for genic sites, or the nearest gene promoter for intergenic regions. These were then matched by gene symbol to the corresponding transcript. For *cis*-correlations, we performed a Pearson correlation between the DNAm levels and transcripts levels. In cases where a DMR paired to multiple transcript variants, we retained only the DMR-mRNA pair that had the strongest *cis*-correlation such that each DMR was paired to only a unique mRNA. Following this, we used Pearson correlations to relate the expression levels with the corresponding variable, i.e., age, BW0, or median lifespan.

**References**

1. Roy S, Sleiman MB, Jha P, et al. Gene-by-environmental modulation of longevity and weight gain in the murine BXD family. *bioRxiv.* 2019:776559.

2. FastQC. <https://www.bioinformatics.babraham.ac.uk/projects/fastqc/>.

3. Langmead B, Trapnell C, Pop M, Salzberg SL. Ultrafast and memory-efficient alignment of short DNA sequences to the human genome. *Genome biology.* 2009;10(3):R25.

4. Li H, Handsaker B, Wysoker A, et al. The Sequence Alignment/Map format and SAMtools. *Bioinformatics.* 2009;25(16):2078-2079.

5. Lassmann T, Hayashizaki Y, Daub CO. SAMStat: monitoring biases in next generation sequencing data. *Bioinformatics.* 2011;27(1):130-131.

6. Lienhard M, Grimm C, Morkel M, Herwig R, Chavez L. MEDIPS: genome-wide differential coverage analysis of sequencing data derived from DNA enrichment experiments. *Bioinformatics.* 2014;30(2):284-286.

7. Robinson MD, McCarthy DJ, Smyth GK. edgeR: a Bioconductor package for differential expression analysis of digital gene expression data. *Bioinformatics.* 2010;26(1):139-140.

8. Ashbrook DG, Arends D, Prins P, et al. The expanded BXD family of mice: A cohort for experimental systems genetics and precision medicine. *bioRxiv.* 2019:672097.

9. Heinz S, Benner C, Spann N, et al. Simple combinations of lineage-determining transcription factors prime cis-regulatory elements required for macrophage and B cell identities. *Molecular cell.* 2010;38(4):576-589.
